# Initial Experience of Contact Laser Vaporization of the Prostate (CVP) for Benign Prostate Hyperplasia Patients With Hemorrhagic Risk

**DOI:** 10.1155/aiu/6108816

**Published:** 2024-12-19

**Authors:** Yushi Araki, Takashi Kawahara, Teppei Takeshima, Kazuhide Makiyama, Hiroji Uemura

**Affiliations:** ^1^Departments of Urology and Renal Transplantation, Yokohama City University Medical Center, Yokohama, Japan; ^2^Department of Urology, Yokohama City University Graduate School of Medicine, Yokohama, Japan

**Keywords:** benign prostate hyperplasia, BPH, contact laser vaporization of the prostate, CVP

## Abstract

**Introduction:** Since contact laser vaporization of the prostate (CVP) was approved by the Japanese insurance system in 2016, the use of a 980 nm diode laser system for CVP has become widespread for treating benign prostate hyperplasia (BPH) patients. Our institute has been implementing CVP for BPH since 2018, treating a total of 93 patients, including 28 with a risk of hemorrhage. This study examines the safety and efficacy of CVP treatment for BPH patients with a hemorrhagic risk.

**Patient and Methods:** A total of 93 BPH patients with lower urinary tract symptoms (LUTS) underwent CVP between February 2018 and September 2022. All patients were on medications for BPH and were refractory to these medications. The median (mean ± SD) age was 72 (72.9±6.27), and the prostate volume was 64 (68.9±32.5). IPSS, QOL index, and OABSS scores for patients not requiring catheterization were 22 (22.1±6.38), 5 (5.24±0.74), and 6 (7±3.29), respectively. The CVP treatment was performed using a 980 nm diode laser. Of the 93 patients, 28 (30.1%) had a hemorrhage risk. This group included 13 (14.0%) who were continuously receiving anticoagulant and/or antiplatelet agents, 13 (14.0%) who temporarily stopped these medications, and 2 (2.1%) who had a hemorrhage risk due to low platelet counts (< 5.0 × 10^4^/*μ*L).

**Results:** Postoperatively, 15 (16.1%) patients, including 11 who were catheterized preoperatively, needed temporary catheterization after CVP treatment. Of these, 14 had their catheters successfully removed. The IPSS score significantly decreased from 22 (22.1±6.38) to 8 (9.02±6.07) (*p* < 0.0001). In patients with hemorrhagic risk on anticoagulant and/or antiplatelet agents, the change in hemoglobin level before and after surgery was 0.6 g/dL, a difference that was not significant. Two of the 15 patients with hemorrhagic risk experienced hemorrhage 23 and 26 days postoperatively and underwent transurethral coagulation. Other perioperative complications classified as Clavien–Dindo Grade 2 or higher occurred in 4 (4.3%) patients.

**Conclusion:** CVP treatment appears to be acceptable for BPH patients with hemorrhagic risk. In this study, late-onset hemorrhage occurred approximately 1 month postoperatively. Close postoperative follow-up is required.

## 1. Introduction

Benign prostate hyperplasia (BPH) is a major risk factor for male lower urinary tract symptoms (mLUTS) and decreases QOL [1]. Since the contact laser vaporization of the prostate (CVP) treatment for BPH was approved by the Japanese insurance system in 2016, CVP using a 980 nm diode laser system has emerged as a potential treatment option for BPH, alongside transurethral resection of the prostate (TUR-P), holmium laser enucleation of the prostate (HoLEP), photoselective vaporization of the prostate (PVP), and transurethral enucleation with a bipolar system (TUEB) [2]. TUR-P is associated with the risk of TUR syndrome, stemming from the use of nonelectrolyte perfusate, and HoLEP requires significant time for technical mastery [3].

CVP is considered an easier technique and has demonstrated efficient vaporization with less hemorrhage [3, 4]. Higher hemostatic capacity and tissue transpiration effect due to the diode laser's 980 nm wavelength, which is absorbed by both water molecules and hemoglobin [3]. In addition, as the population ages, more patients are receiving antithrombotic therapy for coronary artery disease, arrhythmias, and strokes, and withdrawal of antithrombotic drugs may increase the risk of thrombosis, whereas continuation of these drugs may increase the risk of perioperative bleeding. Prior studies have shown safety of continued antithrombotic therapy in HoLEP and PVP [5, 6]. To date, no study has specifically addressed CVP treatment in patients with hemorrhagic risk, including those continuing anticoagulation therapy, temporarily stopping anticoagulation agents, or with conditions that predispose them to hemorrhage. This study examines the safety and efficacy of CVP treatment in BPH patients with a hemorrhagic risk.

## 2. Patients and Methods

A total of 93 patients underwent CVP for BPH from February 2018 to September 2022 at Yokohama City University Medical Center (Yokohama, Japan). This study was approved by the Institutional Review Board of Yokohama City University Medical Center (IRB No. B200600021). Written informed consent was waived due to the retrospective observational nature of the study, and all methods complied with the Declaration of Helsinki. All patients received an alpha-blocker medication and were resistant to this medication. Among these patients, 28 of 93 (30.1%) had a hemorrhage risk, including 13 (14.0%) who continuously received anticoagulant and/or antiplatelet agents, 13 (14.0%) who temporarily stopped them, and 2 (2.1%) with a hemorrhage risk due to low platelet counts (< 5.0 × 10^4^/*μ*L). The detailed anticoagulant and/or antiplatelet agents were as follows including duplicates; Aspirin (Bayaspirin): 11 cases, Clopidogrel: 5 cases, Prasugrel: 1 case, Cilostazol: 2 cases, Limaprost alfadex: 2 cases, Apixaban: 3 cases, Edoxaban: 2 cases, Rivaroxaban: 2 cases.

Preoperative examination included IPSS, QOL-index, OABSS, PSA, and prostate volume using ultrasound (US) or MRI. Patients with a serum PSA level of 4 ng/mL or higher underwent MRI and a free-to-total PSA ratio assessment. When MRI could not rule out prostate cancer, preoperative prostate needle biopsy or prostate needle biopsy at the time of CVP was performed. In this study, we assessed urinary symptoms by the IPSS, QoL index score, and OABSS. The IPSS is most commonly used as an assessment tool for men with BPH, OAB, or who have undergone radical prostatectomy or prostatic radiotherapy [7–10]. The IPSS questionnaire comprises seven questions on LUTS (incomplete emptying, frequency, intermittency, urgency, weak stream, straining, and nocturia) and an additional question to yield a QoL index, scored from zero (delighted) to six (terrible), which was used as the QoL surrogate indicator in this study. OAB is defined as a symptom syndrome that includes urgency, with or without urgency incontinence, and usually with frequency and nocturia [11]. The OABSS, originally developed in Japan, is a 4-item questionnaire to express OAB symptoms on a single scale [12]. The OABSS question items address individual symptoms: daytime frequency, nighttime frequency, urgency, and urgency incontinence. Gotoh et al. reported that OABSS is a useful tool for assessing the effects of treatment on OAB symptoms and responsive to treatment-related changes [13].

**Figure 1:**
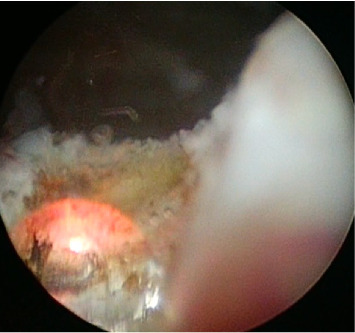
Diode laser is used to proceed with vaporization.

A 980 nm diode laser was used for BPH treatment. Vaporization was conducted mainly from 5:00 to 7:00 o'clock, and finally from 3:00 to 9:00 o'clock (Figure 1). The laser settings started from 100 W and were gradually increased to 150 W. The total energy was restricted to up to 5,00,000 J due to laser fiber limitations. A 20Fr urethral catheter was inserted at the conclusion of the CVP treatment and usually removed 2-3 days postoperatively. All of our patients are given postoperative bladder irrigation until the next morning. In cases of temporary urinary retention postoperatively, a 14Fr urethral catheter was reinserted and removed at the next scheduled hospital visit.

This study examined operative time, the ratio of vaporization time to total operation time, hemoglobin change, perioperative complications, and changes in questionnaire scores including IPSS in BPH patients with high or no hemorrhage risk. Perioperative complications were classified using the Clavien–Dindo classification.

### 2.1. Statistical Analyses

The patients' characteristics and scores were analyzed using the Mann–Whitney U test, one-factor analysis of variance (ANOVA), and chi-square test with the GraphPad Prism software program (GraphPad Software, La Jolla, CA, USA). *p* values of < 0.05 were considered statistically significant.

## 3. Results

### 3.1. Patients' Background

Patients' background information is presented in Table 1. The median (mean ± SD) age was 72 (72.9±6.27).

### 3.2. Surgical Procedure

In terms of the surgical procedure, the operation time was positively correlated with prostate volume, but there was no significant correlation between the duration of the surgical procedure and the overall operation time. Conversely, the percentage of vaporization time in the total operation time was positively correlated with the number of surgical procedures. These data suggest that while urologists can easily acquire the CVP procedure, a greater number of experiences contribute to more effective vaporization.

#### 3.2.1. Peri/Postoperative Outcomes

**Figure 2:**
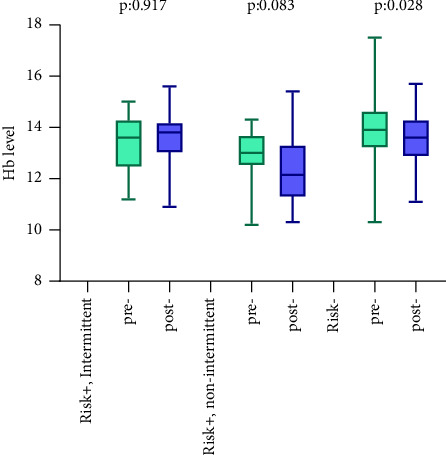
Pre- and postoperative Hb change in each risk group.

The peri/postoperative outcomes are detailed in Table 2. In this study, 28 of 93 (30.1%) patients had a hemorrhage risk, including 13 (14.0%) who were continuously receiving anticoagulant and/or antiplatelet agents, 13 (14.0%) who temporarily stopped them, and 2 (2.1%) with a hemorrhage risk due to low platelet counts (< 5.0 × 10^4^/*μ*L). The indications for antithrombotic medication included angina angiitis, myocardial infarction, atrial fibrillation, and arteriosclerosis. The difference between post- and pre-CVP hemoglobin levels was 0.3 g/dL, and none of the patients required transfusion. In patients with hemorrhagic risk on anticoagulant and/or antiplatelet agents, the change in hemoglobin level was 0.6 g/dL, a difference that was not significant (Figure 2). Two cases (2.2%) underwent transurethral coagulation (TUC) 23 and 26 days postoperatively. These two patients were stopped anticoagulant agents (Edoxaban for atrial fibrillation in case 1. Clopidogrel Sulfate and Rivaroxaban for angina pectoris and atrial fibrillation in case 2) before CVP. They showed no postoperative hematuria and removed urethral catheter 3 days after surgery and discharged hospital 4 days after surgery. But they complained delayed bleeding 23 and 26 days after surgery.

**Figure 3:**
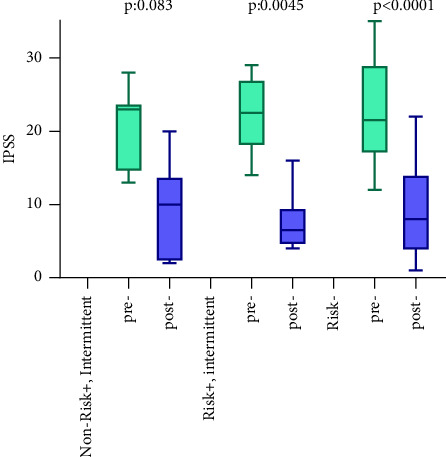
Pre- and postoperative IPSS score in each risk group.

Preoperative IPSS, QOL-index, OABSS, prostate volume, and PSA were 22 (22.1 ± 6.38), 5 (5.24 ± 0.74), 6 (7 ± 3.29), 64 (68.9 ± 32.5) mL, and 4.3 (5.68 ± 5.12) ng/mL, respectively. The postoperative IPSS, measured two or three months postoperatively, was 8 (9.02 ± 6.07). The postoperative IPSS was significantly lower than the preoperative one (*p* < 0.0001) (Figure 3).

Other complications classified as Clavien–Dindo Grade 2 or higher included urinary tract infections (Grade 2) in 3 cases and stroke (Grade 2) in 1 case. The stroke case was thought that this was not an effect of CVP, since no anticoagulants were withdrawn, including during the perioperative period, and relatively many intravenous infusions were also used. Fifteen (16.1%) patients, including 11 who were catheterized preoperatively, had to be catheterized temporarily after CVP treatment [Table 3]. Of these, 14 were successfully catheter removal without additional invasive surgery, except one case who died from liver dysfunction resulting from liver cirrhosis during follow-up.

## 4. Discussion

BPH is defined as a noncancerous increase in the size of the prostate gland and commonly presents with LUTS [14]. BPH with male LUTS significantly affects daily quality of life [1]. Oral medications, including alpha-1 blockers or dutasteride, are widely used, and many patients are able to be managed without surgical procedures [1]. However, some patients still need to undergo endoscopic surgery to control male LUTS or urinary retention.

TUR-P has been a standard procedure for BPH for several decades. Despite its effectiveness, TUR-P has side effects, including the risk of TUR syndrome from using nonelectrolyte irrigation solutions, and a higher risk of hemorrhage compared to HoLEP and vaporization therapy [1].

In the early 1990s, Nd:YAG laser vaporization using a 1,064 nm wavelength was initially introduced for BPH treatment, but this treatment often resulted in severe postoperative edema and urinary retention [15]. In the late 1990s, HoLEP using Ho:YAG laser was introduced, followed by PVP being approved in the Japanese insurance system in 2004 [16, 17]. Diode laser treatment for BPH was first reported in 1996 using 10W low-energy devices. After several improvements, CVP using diode laser treatment was approved in the Japanese insurance system in 2016, and its effectiveness for BPH has been reported recently.

Due to an aging society, anticoagulant medications have become widespread to protect against stroke or acute myocardial infarction [5]. Previous studies have shown the safety of HoLEP and PVP with continuous use of anticoagulant agents [6]. To date, no study has reported on the effectiveness and safety of CVP in BPH patients with hemorrhage risk. This study examined a total of 28 (30.1%) patients with high hemorrhage risk. In the present study, postoperative hemoglobin level fluctuations were minor in patients with and without hemorrhagic risk, and no cases required blood transfusions or perioperative TUC. In these patients, two cases experienced postoperative hemorrhage 23 and 26 days postoperatively and underwent TUC. These two cases were continuously medicated with anticoagulant or antiplatelet agents. Since the possibility of secondary bleeding requiring hospitalization has been reported to occur in any BPH surgery, regardless of the procedure, further studies are needed to determine whether antithrombotic therapy is an independent risk factor in secondary bleeding after CVP [18].

In conclusion, CVP is a safe and efficient procedure that can be performed in patients at risk for bleeding without increasing the risk of perioperative bleeding or complications. On the other hand, we should be aware of the possibility of secondary bleeding in some patients, and we should remind that if patients showed no gross hematuria, a follow-up is needed. In addition, this study was conducted at a single institution with a small; we should remind that if patients showed no gross hematuria, a follow-up is needed. Also, this is a retrospective study of a small population conducted at a single institution, and we await the accumulation of future large-scale clinical trials.

## 5. Conclusion

CVP treatment appears to be acceptable for BPH patients with hemorrhagic risk. In this study, late-onset hemorrhage occurred approximately 1 month postoperatively. Close postoperative follow-up is required.

## Figures and Tables

**Table 1:** Patient characteristics.

	All patients (*n* = 93)	Hemorrhage risk (+) continuatio*n* (+) (*n* = 15)	Hemorrhage risk (+) continuatio*n* (−) (*n* = 13)	Hemorrhage risk (−) (*n* = 65)	*p *value
Age	72 (54–88)	72 (67–81)	74 (66–78)	72 (54–88)	0.837
Prostate volume, cm^3^	64 (20–177)	63 (20–150)	76 (20–177)	62 (20–145)	0.384
PSA, ng/mL	4.3 (0.1–36.0)	4.3 (0.3–12.1)	4.1 (0.4–25.9)	4.3 (0.1–36.0)	0.731
Catheterization (%)	31 (33.3)	7 (46.7)	6 (46.2)	18 (27.7)	0.203
Preoperative					
IPSS	22 (12–35)	23 (13–28)	22.5 (14–29)	22.5 (12–35)	0.535
QOL-index	5 (4–6)	5 (4–6)	5.5 (4–6)	5 (4–6)	0.822
OABSS	6 (2–15)	6 (4–12)	5 (2–13)	7 (2–15)	0.824

**Table 2:** Peri/postoperative outcomes.

	All patients (*n* = 93)	Hemorrhage risk (+) continuatio*n* (+) (*n* = 15)	Hemorrhage risk (+) continuatio*n* (−) (*n* = 13)	Hemorrhage risk (−) (*n* = 65)	*p* value
Operation					
Operation time, min	77 (38–154)	72 (38–116)	80 (57–117)	77 (45–154)	0.970
Laser treatment time, min	51 (19–106)	53 (20–70)	53 (34–85)	48 (19–106)	0.167
Laser energy, KJ	415 (120–740)	436 (120–604)	440 (265–720)	408 (150–740)	0.756
Changes in hemogrobin level, g/dL	0.3 (−1.9 to 1.8)	0.6 (−1.9 to 1.8)	0.0 (−0.9 to 0.9)	0.3 (−1.1 to 1.7)	0.550
Catheterization period, day	3 (1–7)	3 (2–7)	2 (1–4)	2 (1–6)	0.333
Hospital stay period, day	4 (2–10)	4 (3–10)	4 (2–5)	4 (2–8)	0.140
Re-catheterization, n (%)	15 (16.1)	4 (26.7)	2 (15.4)	9 (13.8)	0.364
Complications, *n* (%)					
Perioperative complication	4 (4.3)	1 (6.7)	0 (0.0)	3 (3.8)	0.821
Postdischarge hemorrhage	2 (2.2)	2 (13.3)	0 (0.0)	0 (0.0)	0.030
Postoperative IPSS	8 (1–22)	10 (2–20)	6.5 (4–16)	8 (1–22)	0.738

**Table 3:** Complications.

	Clavien–Dindo grade	Hemorrhage risk (+) continuation (+)	Hemorrhage risk (+) continuation (−)	Hemorrhage risk (−)
Urinary tract infections	2	1	0	2
Stroke	2	0	0	1

## Data Availability

Due to ethical restrictions, the raw data underlying this paper are available upon request to the corresponding author.

## References

[1] Lee S. W., Choi J. B., Lee K. S. (2013). Transurethral Procedures for Lower Urinary Tract Symptoms Resulting from Benign Prostatic Enlargement: A Quality and Meta-Analysis. *Int Neurourol J*.

[2] Kawamura Y., Tokunaga M., Hoshino H., Matsushita K., Terachi T. (2015). Clinical Outcomes of Transurethral Enucleation With Bipolar for Benign Prostatic Hypertrophy. *Tokai Journal of Experimental & Clinical Medicine*.

[3] Shaker H. S., Saafan A., Yassin M. M., Fawzy M., Shoeb M. S. (2015). Safety and Efficacy of Quartz Head Contact Laser Ablation for Large Prostates Using 980-nm Laser: A Comparative Prospective Study against that for Small- and Medium-Sized Prostates. *Urology*.

[4] Miyazaki H., Hirano Y., Kato S. (2018). Early Experiences of Contact Laser Vaporization of the Prostate Using the 980 Nm High Power Diode Laser for Benign Prostatic Hyperplasia. *Lower Urinary Tract Symptoms*.

[5] Ding M., Fratiglioni L., Johnell K., Fastbom J., Ljungdahl M., Qiu C. (2017). Atrial Fibrillation and Use of Antithrombotic Medications in Older People: A Population-Based Study. *International Journal of Cardiology*.

[6] Elzayat E., Habib E., Elhilali M. (2006). Holmium Laser Enucleation of the Prostate in Patients on Anticoagulant Therapy or With Bleeding Disorders. *The Journal of Urology*.

[7] Barry M. J., Williford W. O., Fowler F. J., Jones K. M., Lepor H. (2000). Filling and Voiding Symptoms in the American Urological Association Symptom Index: the Value of Their Distinction in a Veterans Affairs Randomized Trial of Medical Therapy in Men with a Clinical Diagnosis of Benign Prostatic Hyperplasia. *The Journal of Urology*.

[8] Chapple C., Herschorn S., Abrams P., Sun F., Brodsky M., Guan Z. (2009). Tolterodine Treatment Improves Storage Symptoms Suggestive of Overactive Bladder in Men Treated with Alpha-Blockers. *European Urology*.

[9] Moore K. N., Valiquette L., Chetner M. P., Byrniak S., Herbison G. P. (2008). Return to Continence after Radical Retropubic Prostatectomy: A Randomized Trial of Verbal and Written Instructions versus Therapist-Directed Pelvic Floor Muscle Therapy. *Urology*.

[10] Keyes M., Miller S., Moravan V. (2009). Predictive Factors for Acute and Late Urinary Toxicity After Permanent Prostate Brachytherapy: Long-Term Outcome in 712 Consecutive Patients. *International Journal of Radiation Oncology, Biology, Physics*.

[11] Abrams P., Cardozo L., Fall M. (2003). The Standardisation of Terminology in Lower Urinary Tract Function: Report From the Standardisation SubCommittee of the International Continence Society. *Urology*.

[12] Homma Y., Yoshida M., Seki N. (2006). Symptom Assessment Tool for Overactive Bladder Syndrome--Overactive Bladder Symptom Score. *Urology*.

[13] Gotoh M., Homma Y., Yokoyama O., Nishizawa O. (2011). Responsiveness and Minimal Clinically Important Change in Overactive Bladder Symptom Score. *Urology*.

[14] Takada J., Honda N., Hazama H., Ioritani N., Awazu K. (2016). Analysis of Thermally Denatured Depth in Laser Vaporization for Benign Prostatic Hyperplasia Using a Simulation of Light Propagation and Heat Transfer (Secondary Publication). *Laser Therapy*.

[15] Costello A. J., Bowsher W. G., Bolton D. M., Braslis K. G., Burt J. (1992). Laser Ablation of the Prostate in Patients With Benign Prostatic Hypertrophy. *British Journal of Urology*.

[16] Gilling P. J., Kennett K., Das A. K., Thompson D., Fraundorfer M. R. (1998). Holmium Laser Enucleation of the Prostate (HoLEP) Combined With Transurethral Tissue Morcellation: An Update on the Early Clinical Experience. *Journal of Endourology*.

[17] Malek R. S., Barrett D. M., Kuntzman R. S. (1998). High-power Potassium-Titanyl-Phosphate (KTP/532) Laser Vaporization Prostatectomy: 24 hours Later. *Urology*.

[18] Yee C. H., Wong J. H., Chiu P. K. (2016). Secondary Hemorrhage after Bipolar Transurethral Resection and Vaporization of Prostate. *Urology Annals*.

